# What You Should Know About Vasitis: A Case Report

**DOI:** 10.7759/cureus.58785

**Published:** 2024-04-22

**Authors:** Abdullah Ayed, Abdullah M Alshahrani

**Affiliations:** 1 Department of Surgery, College of Medicine, University of Bisha, Bisha, SAU; 2 Department of Family and Community Medicine, College of Medicine, University of Bisha, Bisha, SAU

**Keywords:** vasitis, vasitis nodosa, inguinal hernia, inflammation, vas deferens

## Abstract

Even though infected vasitis is rarely reported in the literature, there are other diagnoses that share the same clinical signs, including testicular torsion, epididymo-orchitis, epididymitis, trauma, and incarcerated hernia. A 27-year-old man was brought to the emergency department by his brother with right inguinal and testicular pain for one day. The history was not significant with fever, lower urinary tract symptoms, urethral discharge, change in bowel habits, previous history of inguinal swelling, or surgical intervention. On presentation, the patient was vitally stable, and right infra-inguinal and inguinal vas deferens were tender and swollen; however, both testes and epididymis were normal, and no urethral discharge. Vasitis, or inflammation of the vas deferens, is an uncommon illness that Chan PT and Schlegel classified as either asymptomatic vasitis nodosa or severely painful infectious vasitis. Acute infective vasitis is a really uncommon illness, with only a few occurrences documented in the literature. However, the retrograde transmission of urinary pathogens such as *Escherichia coli* and *Haemophilus influenza* is thought to cause acute vasitis. Because of its rarity and ambiguous imaging findings, diagnosing vasitis can be difficult. Epididymitis, orchitis, and testicular torsion can all be ruled out with ultrasound and duplex Doppler screening. Inguinal hernia is difficult to distinguish from vasitis with ultrasound; hence, CT and MRI are more commonly used to confirm the diagnosis. Since this is the first occurrence in our city that we are aware of, it was reported. A few cases from Saudi Arabia have also been documented, and by doing so, we may raise clinicians' awareness of this disease and ensure that they can treat patients without making an incorrect diagnosis.

## Introduction

Despite the rarity of reporting infected vasitis in the literature, there are other differential diagnoses that have the same clinical symptoms such as testicular torsion, epididymo-orchitis, epididymitis, trauma, and incarcerated hernia. The clinical symptoms and ultrasonographic features can make it difficult to distinguish vasitis from an incarcerated inguinal hernia because both illnesses induce groin lumps and pain [1]. In general, the cause of the pain and swelling in the scrotum can frequently be determined through physical examination, ultrasonography, and urinalysis. Moreover, lab findings for the white blood count were normal or slightly elevated. Even if a few white blood cells can be found in urine, urine cultures frequently produce disappointing negative results [2].

## Case presentation

A 27-year-old man was brought to the emergency department by his brother with right inguinal and testicular pain for one day. He reported significantly worsening pain with time; his medical history was significant only with gonococcal urethritis three weeks ago, which was treated with a single dose of 125 mg of intramuscular ceftriaxone and a single 1 g dose of oral azithromycin. He had no history of recent trauma. Moreover, the history was not significant with fever, lower urinary tract symptoms, urethral discharge, change in bowel habits, previous history of inguinal swelling, or surgical intervention. The patient is not married, denied any sexual activity, and worked as a teacher.

The patient was seen in an acute care unit with attending staff in full personal protective equipment. On presentation, the patient was vitally stable, and the left infra-inguinal and inguinal vas deferens were tender and swollen; however, both testes and epididymis were normal, and no urethral discharge.

**Figure 1:**
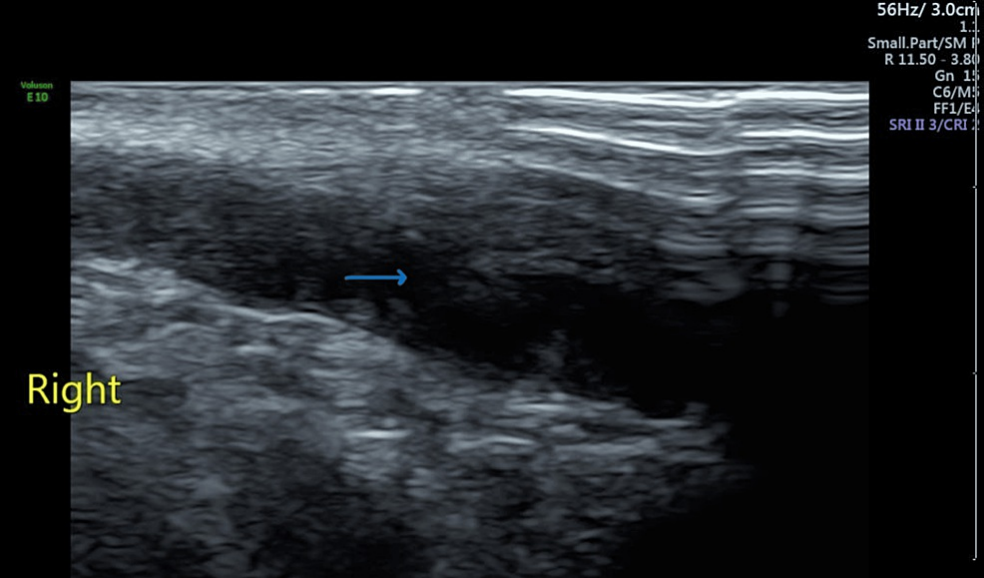
Sagittal section of right inguinal canal showing enlarged vas deferens

**Figure 2:**
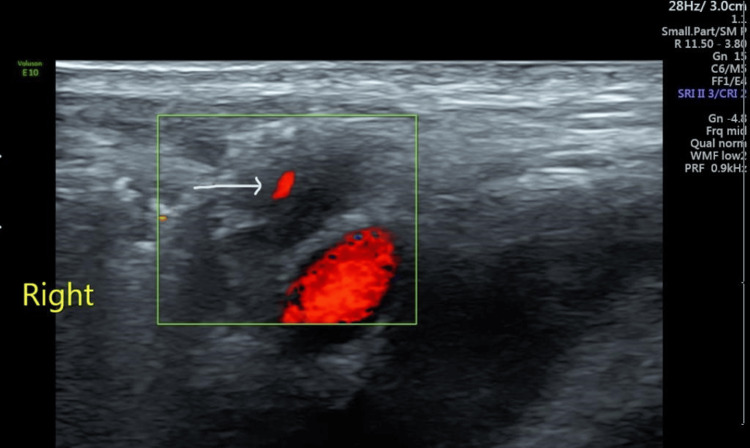
Transverse image showing enlarged right vas deferens with increased vascularity

The patient’s full blood count was within normal range, the urethral swab was negative, and both urinalysis and culture were negative. The tests for liver function and renal profile were normal. The inguinal canal ultrasound was then ordered, with the result revealing enlargement of the right vas deferens (Figure 1) with increased vascularity (Figure 2).

Orchitis, epididymitis, testicular torsion, and inguinal hernia were ruled out by these findings. The patient was treated as an outpatient with oral levofloxacin and a non-steroidal anti-inflammatory drug (NSAID) to relieve the pain after reviewing the patient's labs and inguinal canal ultrasound. After two days, the patient was contacted through the telemedicine clinic, and the treatment resulted in a significant reduction in pain; thus, the patient was instructed to finish the antibiotic course and return in one month. After one month, the patient's swollen left vas deferens improved and the pain subsided.

## Discussion

Vasitis, or inflammation of the vas deferens, is an uncommon illness that Chan PT and Schlegel classified as either asymptomatic vasitis nodosa or severely painful infectious vasitis [3]. Vasitis nodosa is a benign chronic inflammation that causes fusiform nodular thickening of the vas deferens, luminal blockage of the vas deferens, higher intraluminal pressures, spermatozoa leaking, granuloma development, and fibrosis. The majority of patients have had a vasectomy or procedures near the vas deferens, such as herniorrhaphy, perianal fistulectomy, or prostatectomy.

Acute infective vasitis is a really uncommon illness, with only a few occurrences documented in the literature. However, the retrograde transmission of urinary pathogens such as *Escherichia coli* and *Haemophilus influenza* is thought to cause acute vasitis. Chlamydia trachomatis and *Mycobacterium tuberculosis* are two more uncommon pathogens that have been discovered. The majority of the individuals with previously reported vasitis have a history of procedures near the affected vas deferens. Other risk factors for acute vasitis have not been attributed to this disorder due to its low prevalence [4].

Localized pain or a palpable mass in the scrotal or inguinal region, along with leukocytosis or fever, are some of the clinical signs. When there is isolated site engagement, it is easy to become confused. Orchitis, epididymitis, testicular torsion, and inguinal hernia are all common differential diagnoses. Because vasitis is treated with medicines and does not require surgery, accurate identification is critical [5,6].

Because of its rarity and ambiguous imaging findings, diagnosing vasitis can be difficult. Epididymitis, orchitis, and testicular torsion can all be ruled out with ultrasound and duplex Doppler screening. Inguinal hernia is difficult to distinguish from vasitis with ultrasound; hence, CT and MRI are more commonly used to confirm the diagnosis [7,8]. Finally, antibiotics and anti-inflammatory medications are effective in the majority of reported cases [3-9].

## Conclusions

Since this is the first occurrence in our city that we are aware of, it was reported. A few cases from Saudi Arabia have also been documented, and by doing so, we may raise clinicians' awareness of this disease and ensure that they can treat patients without making an incorrect diagnosis. Based on the available literature, most of the reported vasitis can be resolved with the use of anti-inflammatories and antibiotics alone.
